# Improved Pitzer activity model for Tc(iv) solubility and hydrolysis in the Tc(iv)–Na^+^–K^+^–Ca^2+^–Mg^2+^–H^+^–Cl^−^–OH^−^–H_2_O(l) system

**DOI:** 10.1039/d5ra04721h

**Published:** 2025-10-10

**Authors:** C. Kiefer, D. Fellhauer, M. Altmaier, X. Gaona

**Affiliations:** a Karlsruhe Institute of Technology, Institute for Nuclear Waste Disposal Karlsruhe Germany christian.kiefer2@kit.edu xavier.gaona@kit.edu

## Abstract

This study presents improved chemical, thermodynamic and Pitzer activity models for the system Tc(iv)–Na^+^–K^+^–Ca^2+^–Mg^2+^–H^+^–Cl^−^–OH^−^–H_2_O(l) in the context of the German Thermodynamic Reference Database (THEREDA, https://www.thereda.de). The work is based on (i) the previous THEREDA release No. 10 for Technetium in 2016, (ii) the data selection in the second update book of the Nuclear Energy Agency-Thermochemical Database (NEA-TDB) project and (iii) available new solubility studies in dilute to concentrated NaCl, KCl, CaCl_2_ and MgCl_2_ solutions. Updated chemical and thermodynamic models are evaluated to include the hydrolysis species Tc_2_O_3_^2+^, TcO(OH)_2_(aq), TcO(OH)_3_^−^, Ca_3_[TcO(OH)_5_]^3+^ and Mg_3_[TcO(OH)_5_]^3+^. The model provides a new hydrolysis constant for TcO(OH)_3_^−^ and new Pitzer activity coefficient for the interactions between TcO(OH)_3_^−^ and Na^+^/K^+^. The updated model properly describes Tc(iv) solubility data in binary NaCl, KCl, CaCl_2_ and MgCl_2_ salt systems, as well as in selected mixed solutions (ternary, quaternary, quinary). The incorporation of the updated model in thermodynamic databases represents an important tool for source term estimations and speciation calculations of Tc(iv) under a variety of geochemical conditions of relevance in the context of nuclear waste disposal, with focus on high ionic strength conditions.

## Abstract 2

The study provides an extended and improved Pitzer model for Tc(iv) in NaCl, KCl, CaCl_2_ and MgCl_2_ systems, which can be implemented in geochemical calculations of relevance in the context of nuclear waste disposal.

## Graphical abstract

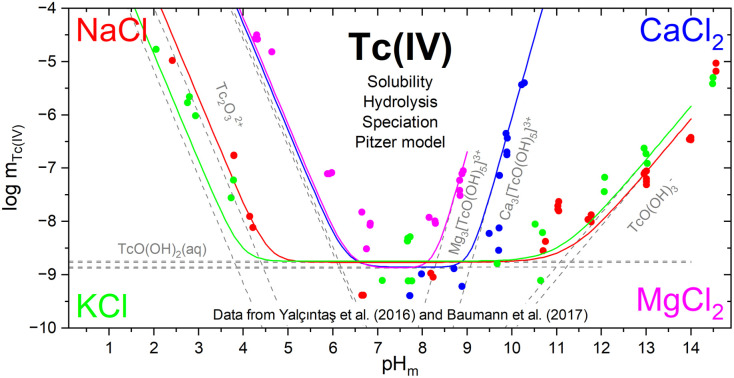

## 1. Introduction

Technetium is a relevant radionuclide in the context of nuclear waste disposal, due to the high-yield production of the long-lived (*t*_1/2_ = 2.11 × 10^5^ a), β-emitting ^99^Tc isotope by fission of ^235^U or ^239^Pu in nuclear reactors. The aquatic chemistry of Tc is characterized by its different oxidation states, of which +VII is predominant under weakly reducing to oxidizing conditions in the form of the highly soluble and mobile pertechnetate anion (TcO_4_^−^). In contrast, Tc(iv) is the predominant oxidation state under the strongly reducing conditions foreseen in deep underground repositories for nuclear waste. Tc(iv) (and other metals in the +IV oxidation state, *e.g.* actinides, Zr, Sn) is characterized by a strong hydrolysis and the formation of sparingly soluble hydrous oxides TcO_2_·*x*H_2_O(s), which are expected to control Tc(iv) solubility over a broad range of pH.^1,2^ Geochemical conditions in deep underground repositories are depending on the repository concept, *e.g.* the selected host-rock, groundwater conditions, waste containers, backfilling material or the waste form/type itself. In rock-salt formations or in certain specific clay systems the formation of brine systems with high concentrations of Na^+^, K^+^, Mg^2+^, Ca^2+^ and Cl^−^ (among others) needs to be taken into account.^3–6^ In order to provide accurate predictions of the chemical behaviour of radionuclides in these systems, dedicated experimental studies and the quantitative thermodynamic modelling of radionuclide solubility and speciation at different ionic strength conditions are of greatest importance.

The solubility of Tc(iv) was investigated in a limited number of experimental studies. Eriksen and co-workers^7^ studied the solubility of TcO_2_·*n*H_2_O(s) as function of pH and CO_2_ partial pressure under neutral to alkaline conditions. In the carbonate-free systems, the authors observed a pH-independent solubility within 6 < pH < 9.5 and an increasing solubility in more alkaline conditions. These findings were explained by the predominance of the species TcO(OH)_2_(aq) and TcO(OH)_3_^−^, with their formation reactions TcO_2_·*n*H_2_O(s) ⇌ TcO(OH)_2_(aq) + (*n* − 1) H_2_O(l) and TcO_2_·*n*H_2_O(s) ⇌ TcO(OH)_3_^−^ + H^+^ + (*n* − 2) H_2_O(l), respectively. Liu and co-workers^8^ investigated the solubility of Tc(iv) in acidic to alkaline pH conditions (1 ≤ pH ≤ 12). Their solubility experiments in redistilled water and simulated groundwater are in moderate agreement with data reported by Meyer and co-workers.^9^ Besides the predominance of TcO(OH)_2_(aq) and TcO(OH)_3_^−^ in weakly acidic to hyperalkaline pH conditions, Liu *et al.* reported also the formation of TcO^2+^ and TcO(OH)^+^ at pH < 3. Hess and co-workers^10^ investigated the solubility of TcO_2_·*x*H_2_O(am) in dilute to concentrated HCl (≤6 M) and NaCl (≤5 M) solutions. At pH < 1 and chloride concentrations above 2.5 M, the authors confirmed the predominance of TcCl_4_(aq) and TcCl_6_^2−^ by UV-VIS spectroscopy and proposed the formation of TcCl_4_(s). The increase in solubility observed with increasing NaCl concentration was exclusively attributed to ion interactions between the Tc(iv) species and the background electrolyte. Hess and co-workers performed their experiments from both under- and oversaturation, and observed different pH dependences in the acidic pH range, namely slopes (log *m*_Tc(IV)_*vs.* pH) of −1 and −2, respectively. This behaviour was explained by the formation of the cationic Tc(iv) hydrolysis species TcO(OH)^+^ and TcO^2+^. The predominance of TcO(OH)^+^ over a wide pH-range was later refuted by Yalçintaş,^11^ who showed that their undersaturation solubility experiments also approach a slope of −2 when attaining equilibrium conditions after long equilibration times. The Nuclear Energy Agency followed this argumentation in the second update book on actinides and technetium thermodynamics^2^ released in the context of their Thermochemical Database Project (NEA-TDB) and did not select thermodynamic data for TcO(OH)^+^. Yalçintaş and co-workers^12^ also investigated the solubility of Tc(iv) in dilute to concentrated NaCl, CaCl_2_ and MgCl_2_ solutions. Their undersaturation solubility experiments in 0.1 to 5.0 M NaCl solutions showed enhanced solubility in the acidic (slope = −2) and alkaline (slope = 1) pH region with respect to the solubility minimum in near-neutral conditions, which was explained by the formation of Tc_3_O_5_^2+^ under acidic and TcO(OH)_3_^−^ under alkaline conditions. With increasing ionic strength, the authors observed an increase of solubility in the acidic range and a decrease in alkaline conditions, which were attributed to ionic interactions. Their experiments in 0.25 to 4.5 M CaCl_2_ and MgCl_2_ solutions showed the same pH dependence as in NaCl solutions under acidic conditions, but significantly different behavior in alkaline solutions, namely a strong increase of solubility with a slope of 3 at pH_m_ > 8 (with pH_m_ = –log[H^+^], and [H^+^] in molal units) in 4.5 M MgCl_2_ and pH_m_ > 9, pH_m_ > 10 and pH_m_ > 10.5 in 4.5, 2.0 and 1.0 M CaCl_2_ solutions, respectively. This increase in solubility was explained by the formation of the aqueous complexes Mg_3_[TcO(OH)_5_]^3+^ and Ca_3_[TcO(OH)_5_]^3+^. Based on their experimental data and the NEA-TDB selection,^13^ the authors derived SIT and Pitzer activity models for the system Tc(iv)–Na^+^–Mg^2+^–Ca^2+^–H^+^–Cl^−^–OH^−^–H_2_O(l). Their selected speciation, equilibrium constants and Pitzer parameters were the basis of the THEREDA Tc(iv) release (Release No. 10) in 2016 (see https://www.thereda.de). Baumann and co-workers^14^ performed Tc(iv) undersaturation solubility experiments in 0.1 to 4.0 M KCl solutions. The authors observed a similar behaviour as Yalçintaş and co-workers in the NaCl system, and proposed the same chemical model including Tc_3_O_5_^2+^, TcO(OH)_2_(aq), TcO(OH)_3_^−^ and TcO_2_·0.6H_2_O(am, hyd) as solubility-controlling solid phase. The authors provided as well SIT and Pitzer activity models for the system Tc(iv)–K^+^–Cl^−^–OH^−^–H_2_O(l). In the context of the NEA-TDB project, thermodynamic data available for the solubility and hydrolysis of Tc(iv) were critically reviewed in three different volumes, *i.e.*, volume 3,^1^ volume 5 (ref. 13) and volume 14.^2^ The most recent volume includes the selection of two Tc(iv) hydrous oxide solid phases, TcO_2_(am, hyd, fresh), TcO_2_(am, hyd, aged), and three hydrolysis species, Tc_2_O_2_(OH)_2_^2+^, TcO(OH)_2_(aq) and TcO(OH)_3_^−^.

The present work aims at providing updated and improved chemical, thermodynamic and Pitzer activity models for the system Tc(iv)–Na^+^–K^+^–Mg^2+^–Ca^2+^–Cl^−^–OH^−^–H_2_O(l), considering all experimental solubility data available to date and seeking consistency with the NEA-TDB thermodynamic selection.^2^ The updated Pitzer model is to be implemented in the Release 2025 of the Thermodynamic Reference Database (THEREDA).^6,15^ THEREDA is a database created for geochemical model calculations in the context of nuclear waste disposal focusing on solubility calculations of radionuclides, fission products and waste matrix elements. High saline conditions may be encountered in salt-rock and clay-rock formations under consideration in Germany, and thus ionic strength corrections in THEREDA are accounted for using the Pitzer formalism.^6,15^ As a reference database, THEREDA aims at the consistency, completeness and traceability of the selected data.

## 2. The Pitzer activity model for saline systems with high ionic strength

The Pitzer activity model is based on a set of equations allowing the precise calculation of activity coefficients for single and mixed electrolyte systems up to high ionic strength conditions. For a complete derivation of the equations or an understanding of their physical background the reader is referred to the original publications by Pitzer.^16–21^ The Pitzer equations contain binary parameters describing interactions between each cation–anion pair, *β*^0^_MX_, *β*^1^_MX_, *β*^2^_MX_, *C*^*ϕ*^_MX_, and mixed electrolyte parameters describing interactions between pairs of (unlike) cations or anions, *θ*_Mc_, *θ*_Xa_, cation-cation-anion and anion-anion-cation triplets, *Ψ*_cXa_, *Ψ*_Mca_, as well as parameters for pairs of ions and neutral species, *λ*_nM_ and *λ*_nX_, where M and c are cations, X and a are anions and *n* are neutral species. These so-called Pitzer parameters are specific for pairs/triples of cations, anions and neutral species and empirical values for common ions are often tabulated for example by Harvie *et al.*^22^

## 3. Definition of chemical model and modelling approach

The previous data selection in THEREDA (Release No. 10, 2016) was based on the results of the comprehensive Tc(iv) solubility study by Yalçintaş and co-workers,^12^ conducted in dilute to concentrated NaCl, MgCl_2_ and CaCl_2_ solutions. The thermodynamic data in the second NEA-TDB update book^2^ are based on the work by Yalçintaş *et al.*, but consider also data at low ionic strength conditions reported in previous studies.^7,8^ The study of Baumann and co-workers^14^ investigating Tc(iv) solubility in dilute to concentrated KCl solutions is neither considered in THEREDA (Release No. 10, 2016) nor in the NEA-TDB^2^ compilations, which accordingly do not include (SIT, Pitzer) ion interaction parameters of Tc(iv) species with K^+^. The present work is part of the systematic improvement of THEREDA, and is intended to update the thermodynamic data and Pitzer parameters of the system Tc(iv)–Na^+^–K^+^–Ca^2+^–Mg^2+^–H^+^–Cl^−^–OH^−^–H_2_O(l).

In our work, TcO_2_·0.6H_2_O(am, hyd) is identified as solubility-controlling hydrous oxide phase. It is the same phase as selected in THEREDA (Rel. No. 10, 2016), and equivalent to TcO_2_(am, hyd, aged) in NEA-TDB^2^ holding the same value for the solubility constant. We have retained the number of hydration waters in the formulation of this solid phase, *i.e.* 0.6H_2_O, as this parameter was accurately quantified by Yalçintaş *et al.*^12^ and has an impact on the calculated Tc(iv) solubility in high ionic strength conditions (particularly MgCl_2_ and CaCl_2_) related to the important changes in the activity of water. The related solid phase TcO_2_(am, hyd, fresh) is slightly more soluble (thermodynamically less stable) and is selected in the NEA-TDB to define the redox equilibrium with Tc(vii). It is observed only at very short contact times (*i.e.*, a few hours to a few days)^1,23^ and not expected to control the Tc(iv) solubility in long-term solubility experiments or even under repository conditions. However, for the sake of completeness and for consistency with NEA-TDB, TcO_2_(am, hyd, fresh) is additionally discussed in the thermodynamic data selection in this work. Based on slope analysis of solubility data and the analogy with Mo(iv) and Zr(iv), Yalçintaş *et al.* and THEREDA (Rel. No. 10, 2016) selected the complex Tc_3_O_5_^2+^ (ref. 12) as predominant aqueous species under acidic conditions. The reviewers of the second update volume of NEA-TDB^2^ favored instead the selection of a dimeric species, Tc_2_O_2_(OH)_2_^2+^, based on new spectroscopic evidence. The selection of a dimeric species has accordingly been favored also in this work. However, a chemical model including the related species Tc_2_O_3_^2+^ which we propose here (same charge and same pH dependence as Tc_2_O_2_(OH)_2_^2+^, clear indication for the existence of the Tc_2_O_3_^2+^ moiety available in Kanellakopulos *et al.*^24^) resulted in a better description of solubility data at high ionic strength conditions, again due to the significant contribution of the additional water molecule in Tc_2_O_2_(OH)_2_^2+^. A comparison of the models including different acidic species is show in Fig. SI-1 and Table SI-1. In weakly acidic to weakly alkaline pH conditions, there is clear evidence of the predominance of the neutral complex TcO(OH)_2_(aq). Under alkaline conditions, TcO(OH)_3_^−^ in NaCl and KCl, Ca_3_[TcO(OH)_5_]^3+^ in CaCl_2_ and Mg_3_[TcO(OH)_5_]^3+^ in MgCl_2_ media are the predominant species, respectively. Note that the complexes Ca_3_[TcO(OH)_5_]^3+^ and Mg_3_[TcO(OH)_5_]^3+^ are not selected in the NEA-TDB review due to the very high ionic strength conditions required for their formation in aqueous solutions which is outside the NEA-TDB scope in terms of ionic strength conditions. The absence of these complexes in the chemical model, however precludes an accurate description of Tc(iv) solubility in concentrated MgCl_2_ and CaCl_2_ solutions, and they have therefore been included in the current model update. The relevant solubility equilibria of the selected Tc(iv) species defining our chemical model are summarized in eqn (1)–(5). The 2nd update book of the NEA-TDB^2^ selected three Tc(iv)-chloride aqueous complexes (TcCl_5_^−^, TcCl_6_^2−^ and Tc_2_OCl_10_^4−^) based on available spectroscopic evidence.^25,26^ These complexes have been disregarded in the chemical model selected in this work, as they form only in very acidic (pH < 1.5) solutions with high chloride concentrations, *i.e.*, far away from the typical geochemical conditions of relevance in underground repositories for nuclear waste.

1 2 TcO_2_·0.6H_2_O(am) + 2 H^+^ ⇌ Tc_2_O_3_^2+^ + 2.2H_2_O(l)

2 TcO_2_·0.6H_2_O(am) + 0.4H_2_O(l) ⇌ TcO(OH)_2_(aq)

3 TcO_2_·0.6H_2_O(am) + 1.4H_2_O(l) ⇌ TcO(OH)_3_^−^ + H^+^

4 TcO_2_·0.6H_2_O(am) + 3.4H_2_O(l) + 3Ca^2+^ ⇌ Ca_3_[TcO(OH)_5_]^3+^ + 3H^+^

5 TcO_2_·0.6H_2_O(am) + 3.4H_2_O(l) + 3Mg^2+^ ⇌ Mg_3_[TcO(OH)_5_]^3+^ + 3H^+^

Solubility calculations with the Pitzer parameters selected in the previous THEREDA release (Release No. 10, 2016) for the cationic species under acidic conditions represent well the available experimental data in dilute to concentrated salt systems. Thus, the parameters for the hydrolysis species Tc_2_O_3_^2+^ have been retained.

In the alkaline region, the addition of the datasets of Baumann and co-workers^14^ requires a new determination of the formation constant of the predominant species TcO(OH)_3_^−^ and the determination of Pitzer parameters for the interaction of this species with K^+^. For this purpose, the sum of the squared residuals (SSR) ∑(log[Tc]_exp_ − log[Tc]_calc_)^2^ of the corresponding samples (alkaline NaCl and KCl solutions) was minimized under the variation of log *K*° and *β*^0^, with [Tc]_exp_ representing measured technetium concentrations and [Tc]_calc_ corresponding to calculated technetium concentrations ([Tc]_calc_ = 2 × [Tc_2_O_3_^2+^]_calc_ + [TcO(OH)_2_(aq)]_calc_ + [TcO(OH)_3_^−^]_calc_). Values of SSR and SSR/No (with No corresponding to the number of samples in each system) are provided in the Table SI-2. Due to the limited number of datasets, other Pitzer parameters for these interactions (*β*^1^_MX_ and *C*^*ϕ*^_MX_) were fixed to default values of *β*^1^_MX_ = 0.3 (as suggested estimation by Grenthe *et al.*^27^ based on charge analogies) and *C*^*ϕ*^_MX_ = 0, respectively, to avoid overparameterization. Parameters of other interactions between basic species (Na^+^–K^+^–Ca^2+^–Mg^2+^–H^+^–Cl^−^–OH^−^–H_2_O(l)), *e.g.*, *θ*, *Ψ* or *λ* were used as selected in THEREDA, and are included in Table SI-3 in the SI. The data evaluation considers all experimental datasets available in dilute systems and with NaCl/KCl as background electrolyte solutions.^7,8,12,14^

Pitzer coefficients for Ca_3_[TcO(OH)_5_]^3+^ and Mg_3_[TcO(OH)_5_]^3+^ were maintained as reported by Yalçıntaş *et al.*^12^ and selected in the previous THEREDA release (Release No. 10, 2016), whereas equilibrium constants were slightly adjusted to maintain consistency with the solubility product selected in the NEA-TDB for TcO_2_(am, hyd, aged). The validity of this updated model was counterchecked against independent experimental solubility data in mixed salt systems (ternary, quaternary, quinary), as described in Section 4.4.

## 4. Results and discussion

### 4.1 Tc(iv) solubility and hydrolysis in dilute solutions, NaCl and KCl systems

**Fig. 1:**
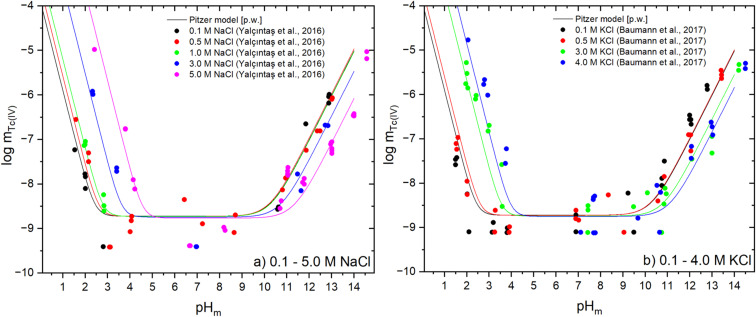
Comparison of experimental solubility data in (a) 0.1 M (0.10 m) to 5.0 M (5.61 m) NaCl solutions from Yalçıntaş *et al.*^12^ and (b) 0.1 M (0.10 m) to 4.0 M (4.58 m) KCl solutions from Baumann *et al.*^14^ with thermodynamic calculations using the chemical, thermodynamic and Pitzer activity models derived in this work (see Tables 1 and 2). Values of Tc and H^+^ concentrations provided in molal units.

The experimental solubility behaviour of Tc(iv) in the absence of a specific background electrolyte and in NaCl and KCl systems is very similar in terms of pH dependency and overall Tc(iv) concentrations. In these systems, an enhanced Tc(iv) solubility is observed under acidic and alkaline conditions due to the predominance of cationic and anionic hydrolysis species, respectively. The minimum of the solubility curve is represented by a pH independent region due to the predominance of equilibrium 2, and ranges from pH_m_ ≈ 3–9 for dilute solutions and pH_m_ ≈ 5–10 for concentrated NaCl and KCl solutions. Note that solubility experiments dealing with sparingly soluble M(IV) (with M = An (Th, U, Np, Pu), Tc, Zr, *etc.*) are characterized by a large dispersion in the pH region where the neutral species M(OH)_4_(aq) or MO(OH)_2_(aq) prevail.^28,29^ The reasons for this are multiple and still under debate: (i) this pH-region is always the one with the lowest solubility, often close to the detection limit of the analytical techniques; (ii) neutral species have a large tendency to sorb on surfaces (vessels' walls, filters, *etc.*); (iii) neutral species have a larger tendency to build colloids; among others. The uncertainties in the experimental data are accordingly reflected in the uncertainties of the corresponding equilibrium constants. Under acidic conditions a strong increase of Tc(iv) solubility with increasing NaCl/KCl concentrations is observed, while the opposite trend is observed under alkaline conditions. This behaviour reflects the ionic interactions of the cationic and anionic Tc(iv) hydrolysis species with chloride and Na^+^/K^+^, respectively. Fig. 1a shows experimental Tc(iv) solubility data of Yalçıntaş *et al.*^12^ in dilute to concentrated NaCl solutions together with solubility calculations using the improved THEREDA model derived in the present work as described in Section 3 and summarized in Tables 1 and 2. Model calculations are in a good overall agreement with experimental data, in spite of the scatter of the latter. The updated model provides also a good description of the Tc(iv) solubility data reported in dilute to concentrated KCl solutions^14^ as shown in Fig. 1b. This is one of the key improvements on the Tc(iv) selection with respect to THEREDA Release No. 10 (2016) and NEA-TDB reviews.

Chemical and thermodynamic models for the Tc(iv)–Na^+^–K^+^–Ca^2+^–Mg^2+^–H^+^–Cl^−^–OH^−^–H_2_O(l) system as selected in the THEREDA Release No. 10 (2016) and improved in this work. All data corresponding to *T* = 298.15 K, *P* = 0.1 MPa and, for aqueous species, infinite dilution (*I* = 0)

**Table:** 

Reaction	THEREDA Release No. 10 (2016), source	THEREDA Release 2025, this work, source
Solubility	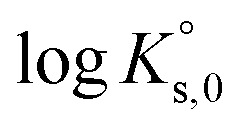	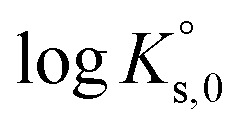
TcO_2_·0.6H_2_O(am) + 0.4 H_2_O(l) ⇌ TcO(OH)_2_(aq)	–(8.80 ± 0.50), Yalçıntaş *et al.*^12^	–(8.72 ± 0.40), Grenthe *et al.*^2^
TcO_2_(am, hyd, fresh) + H_2_O(l) ⇌ TcO(OH)_2_(aq)		–(7.66 ± 1.22),^a^ Grenthe *et al.*^2^

a Solid phase and solubility product selected for consistency with NEA-TDB.^2^ Observed only at very short contact times, and accordingly not expected as solubility-controlling phase in standard solubility experiments or under repository conditions.

b Same log *β° as in Grenthe *et al.*,^2^ but defined for Tc_2_O_3_^2+^ instead of Tc_2_O_2_(OH)_2_^2+^

c Slight changes with respect to Yalçıntaş *et al.*^12^ arising from the slight modification in the solubility product of TcO_2_·0.6H_2_O(am) introduced by Grenthe *et al.*^2^

**Table:** 

Reaction	THEREDA Release No. 10 (2016), source	THEREDA Release 2025, this work, source
Hydrolysis	log *β°	log *β°
3 TcO(OH)_2_(aq) + 2 H^+^ ⇌ Tc_3_O_5_^2+^ + 4 H_2_O(l)	(21.90 ± 0.10), Yalçıntaş *et al.*^12^	
2 TcO(OH)_2_(aq) + 2 H^+^ ⇌ Tc_2_O_3_^2+^ + 3 H_2_O(l)		(12.99 ± 0.41),^b^ present study
TcO(OH)_2_(aq) + H_2_O(l) ⇌ TcO(OH)_3_^−^ + H^+^	–(10.52 ± 0.10), Yalçıntaş *et al.*^12^	–(10.5 ± 0.3), present study
TcO(OH)_2_(aq) + 3 H_2_O(l) + 3 Ca^2+^ ⇌ Ca_3_[TcO(OH)_5_]^3+^ + 3 H^+^	–(32.85 ± 0.30), Yalçıntaş *et al.*^12^	–(32.93 ± 0.30),^c^ present study
TcO(OH)_2_(aq) + 3 H_2_O(l) + 3 Mg^2+^ ⇌ Mg_3_[TcO(OH)_5_]^3+^ + 3 H^+^	–(31.52 ± 0.50), Yalçıntaş *et al.*^12^	–(31.60 ± 0.50),^c^ present study

**Table 2:** Pitzer parameters for the Tc(iv)–Na^+^–K^+^–Ca^2+^–Mg^2+^–H^+^–Cl^−^–OH^−^–H_2_O(l) system as selected in the THEREDA Release No. 10 (2016) and improved in this work

Cation	Anion	*β* ^0^	β^1^	C^ϕ^	Source
**This work (THEREDA Release 2025)**					
Tc_2_O_3_^2+^	Cl^−^	−0.3681	2.6972	0.0063	Yalçıntaş *et al.*^12^
Ca_3_[TcO(OH)_5_]^3+^	Cl^−^	−0.074	4.3	0.015	Yalçıntaş *et al.*^12^
Mg_3_[TcO(OH)_5_]^3+^	Cl^−^	−0.074	4.3	0.015	Yalçıntaş *et al.*^12^
Na^+^	TcO(OH)_3_^−^	0.1085	0.3	0	Present study
K^+^	TcO(OH)_3_^−^	0.1650	0.3	0	Present study
Ca^2+^	TcO(OH)_3_^−^	0.3	1.7	0	Yalçıntaş *et al.*^12^

**THEREDA Release No. 10 (2016)**					
Tc_3_O_5_^2+^	Cl^−^	−0.3681	2.6972	0.0063	Yalçıntaş *et al.*^12^
Ca_3_[TcO(OH)_5_]^3+^	Cl^−^	−0.074	4.3	0.015	Yalçıntaş *et al.*^12^
Mg_3_[TcO(OH)_5_]^3+^	Cl^−^	−0.074	4.3	0.015	Yalçıntaş *et al.*^12^
Na^+^	TcO(OH)_3_^−^	−0.0087	0.3	0.035	Yalçıntaş *et al.*^12^

**Fig. 2:**
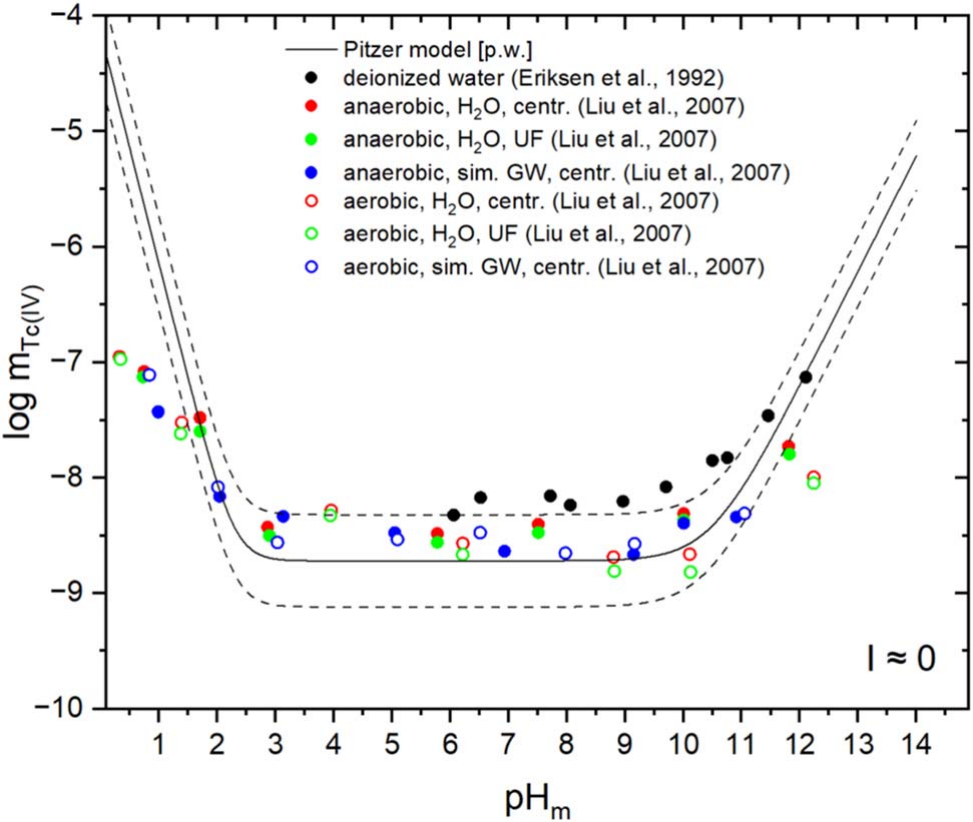
Comparison of experimental solubility data reported by Eriksen *et al.*^7^ in deionized water and Liu *et al.*^8^ in redistilled water or simulated ground water (*I* ≈ 1 mmol L^−1^) with thermodynamic calculations at *I* = 0 using the chemical and thermodynamic models derived in this work (see Table 1). Dashed lines indicate the uncertainty limits of the calculated solubility. Values of Tc and H^+^ concentrations provided in molal units.

Fig. 2 shows the experimental solubility data reported in Eriksen *et al.*^7^ and Liu *et al.*^8^ in deionized water and simulated groundwater, respectively. These datasets were not considered in the previous THEREDA release (Release No. 10, 2016). Due to the low ionic strength of the background electrolytes, these data are compared with model calculation conducted at I = 0, not using Pitzer activity coefficients, to prove the validity of the selected constants for the solid and aqueous species. When considering the corresponding uncertainties (dashed lines in Fig. 2), a moderate to good agreement is obtained between model calculations and experimental datasets. The (slightly) higher solubility data reported by Eriksen and co-workers can be attributed to the use of a less aged solid phase, TcO_2_(am, hyd), in the solubility experiments. The authors report a 
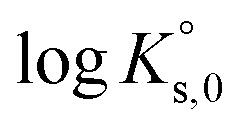
 of −8.16, which lies between our values for fresh (−7.66) and aged (−8.72) solid phases (see Table 1). Therefore, we assume that Eriksen and co-workers used a freshly prepared solid phase, which was subject to ageing processes, but did not reach equilibrium before the experiments were conducted. Solubility data reported by Liu and co-workers are in good agreement with thermodynamic calculations using the updated model derived in this work. Deviations observed in more acidic conditions are attributed to the slow equilibration kinetics observed in this pH region by Hess *et al.*^10^ and Yalçıntaş *et al.*,^12^ and discussed in the NEA-TDB second update book.^2^

### 4.2 Tc(iv) solubility and hydrolysis in dilute to concentrated CaCl_2_ and MgCl_2_ systems

**Fig. 3:**
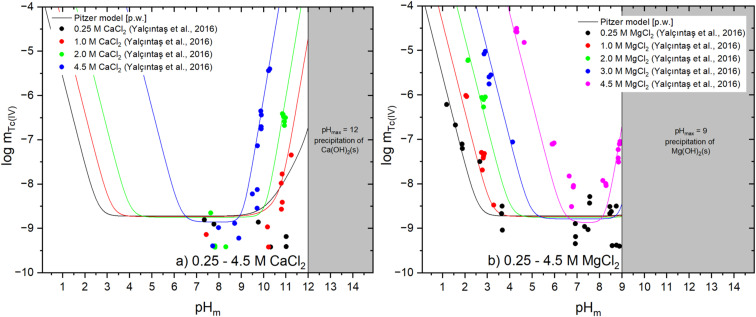
Comparison of experimental solubility data in (a) 0.25 M (0.25 m) to 4.5 M (5.25 m) CaCl_2_ solutions from Yalçıntaş *et al.*^12^ and (b) 0.25 M (0.25 m) to 4.5 M (5.15 m) MgCl_2_ solutions from Yalçıntaş *et al.*^12^ with thermodynamic calculations using the chemical, thermodynamic and Pitzer activity models derived in this work (see Tables 1 and 2). Values of Tc and H^+^ concentrations provided in molal units.

The experimental solubility behavior of Tc(iv) in CaCl_2_ and MgCl_2_ solutions shows some similarities, but also very significant differences to the 1 : 1 background electrolyte systems discussed above. As for NaCl and KCl solutions an increased solubility is observed under acidic and alkaline conditions and a remarkable dispersion in the experimental data especially in near neutral pH conditions. Compared to the Tc(iv) systems in NaCl and KCl solutions, however, the increase in Tc(iv) solubility with pH_m_ is steeper in alkaline CaCl_2_ and MgCl_2_ solutions, and caused by the formation of the ternary complexes Ca_3_[TcO(OH)_5_]^3+^ or Mg_3_[TcO(OH)_5_]^3+^. Under both acidic and alkaline conditions, a strong and systematic increase of Tc(iv) solubility with increasing ionic strength is observed. This behavior is considered in the thermodynamic evaluation particularly by ionic interactions between the predominant cationic Tc(iv) species and chloride ions. No new Tc(iv) solubility studies in these background electrolyte solutions were published since the previous THEREDA release (Release No. 10, 2016) and Second Update book of the NEA-TDB. Accordingly, solubility constants and ion interaction parameters for Ca_3_[TcO(OH)_5_]^3+^ or Mg_3_[TcO(OH)_5_]^3+^ species have been retained as reported by Yalçıntaş *et al.*^12^ The stability constants derived in this work are summarized in Table 1, and refer to an equilibrium with the aqueous master species TcO(OH)_2_(aq). The minor differences with respect to the THEREDA release no. 10 (2016) are mainly related to the slight change in the solubility product of TcO_2_·0.6H_2_O(am) introduced by Grenthe *et al.*^2^ Due to the precipitation of Ca(OH)_2_(s) or Mg(OH)_2_(s) (or corresponding hydroxychloride phases at high chloride concentrations), the solubility data of Tc(iv) in CaCl_2_ and MgCl_2_ solutions are limited to a maximum pH_m_ of ≈ 12 or 9, respectively. Fig. 3a and b show the comparison of experimental Tc(iv) solubility data in dilute to concentrated CaCl_2_ and MgCl_2_ solutions reported by Yalçıntaş *et al.*^12^ and solubility calculations using the thermodynamic and Pitzer activity models updated in this work.

### 4.3 Updated chemical, thermodynamic and Pitzer activity models for the system Tc(iv)–Na^+^–K^+^–Ca^2+^–Mg^2+^–H^+^–Cl^−^–OH^−^–H_2_O(l)

Tables 1 and 2 summarize the chemical, thermodynamic and Pitzer activity models improved in this work. The core of the equilibrium constants remains virtually the same as in Grenthe *et al.*,^2^ with only minor changes in the log *β° for TcO(OH)_3_^−^ resulting from the evaluation of new solubility data not reviewed by the NEA-TDB. The ternary hydrolysis complexes of Tc(iv) with Ca and Mg are also incorporated, as required to describe the solubility of Tc(iv) in concentrated CaCl_2_ and MgCl_2_ solutions. Under such boundary conditions, solubility calculations neglecting the formation of these ternary species will results in a gross underestimation of the actual Tc concentrations. Pitzer coefficients for ionic species selected in the THEREDA Release No. 10 (2016) are retained, with the exception of the interactions of TcO(OH)_3_^−^ with Na^+^ and K^+^, which have been updated and introduced, respectively, on the basis of a new data evaluation including all data available in dilute solutions, as well as NaCl and KCl systems. The updated models are included in the THEREDA Release 2025.

### 4.4 Tc(iv) solubility in simulated reference systems with mixed background electrolytes

The performance of the chemical, thermodynamic and activity models derived in this work has been evaluated against independent Tc(iv) solubility data reported by Baumann and co-workers^14^ in simulated reference systems with mixed background electrolytes of high ionic strength (*I*_m_ > 5.1 mol kg^−1^). The reference systems correspond to two NaCl-dominated cement pore waters as described by Bube *et al.*,^30^ a groundwater of the Canadian Shield crystalline rock^31^ and a simulated WIPP brine.^32^ The compositions of these samples are shown in Table 3.

**Table 3:** Composition of the samples of the simulated reference systems with mixed background electrolytes as reported by Baumann *et al.*^14^

System	[NaCl] [mol kg^−1^]	[KCl] [mol kg^−1^]	[CaCl_2_] [mol kg^−1^]	[MgCl_2_] [mol kg^−1^]	pH_m_
Cement L/ILW simulates exposed to NaCl brine	5.641	0.614			13.33
Cement L/ILW simulates exposed to NaCl brine	4.923	0.240			12.93
Canadian reference groundwater	2.321	0.341	0.851	0.359	7.05
WIPP generic weep brine	3.113	0.437		1.032	8.65

**Fig. 4:**
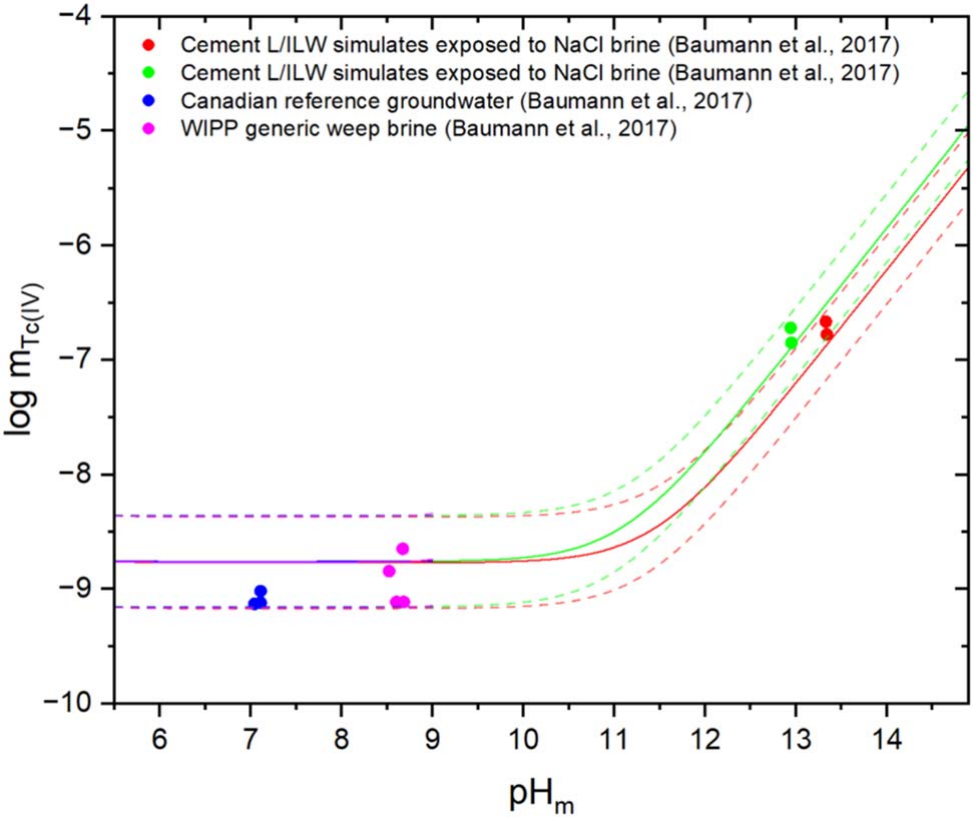
Comparison of experimental solubility data in simulated reference systems with mixed background electrolytes from Baumann *et al.*^14^ (symbols) and thermodynamic calculations using the chemical, thermodynamic and Pitzer activity models derived in this work (solid lines). Dashed lines indicate the uncertainty limits of the calculated solubilities. Values of Tc and H^+^ concentrations provided in molal units.

Fig. 4 shows the Tc(iv) experimental solubility data in the reference systems as reported by Baumann *et al.*^14^ together with thermodynamic calculations conducted with the updated models derived in this work for the different solution compositions. The figure confirms that model calculations are in excellent agreement with independent experimental solubility data for mixed electrolyte systems, thus supporting that thermodynamic and Pitzer activity models derived for simple salt systems (NaCl, KCl, MgCl_2_, CaCl_2_) can be also satisfactorily applied to more complex geochemical systems as expected under repository conditions. Such comparison was included neither in the THEREDA Release No. 10 (2016) nor in the second update book of the NEA-TDB, and thus represents and additional improvement of the current model.

## 5. Summary and conclusions

The present work provides improved chemical, thermodynamic and Pitzer activity models for the system Tc(iv)–Na^+^–K^+^–Ca^2+^–Mg^2+^–H^+^–Cl^−^–OH^−^–H_2_O(l). The work was performed in the context of the systematic improvement of the German Thermodynamic Reference Database (THEREDA). The updated models consider all reliable solubility datasets in NaCl, KCl, CaCl_2_ and MgCl_2_ solutions available to date. Thermodynamic data selection in the THEREDA Release No. 10 (2016) and in the second update book of the NEA-TDB project are taken as anchoring points in the current data evaluation process. The improved model also implements ion interaction processes in dilute to concentrated KCl solutions, which were neither considered in the THEREDA Release No. 10 (2016) nor in the second update book of the NEA-TDB, since data in these systems were not available. The incorporation of the ternary complexes Ca_3_[TcO(OH)_5_]^3+^ or Mg_3_[TcO(OH)_5_]^3+^ (disregarded in the NEA-TDB, because of their presence only in high ionic strengths out of the scope of NEA-TDB) is mandatory for an accurate description of Tc(iv) solubility in concentrated CaCl_2_ and MgCl_2_ systems, respectively. Chemical, thermodynamic and Pitzer activity models derived in this work from binary salt systems (NaCl, KCl, MgCl_2_, CaCl_2_) are compared with independent solubility data in mixed solutions (ternary, quaternary, quinary), indicating the correct performance of the updated models in more complex geochemical boundary conditions as those expected in underground repositories for nuclear waste disposal.

## Conflicts of interest

There are no conflicts to declare.

## Supplementary Material

RA-015-D5RA04721H-s001

## Data Availability

The experimental solubility data used in the model and compared to the model are available in the original publications as cited in the present article. Model parameters used in this article have been included in the article or the supplementary information (SI) and are available at the Thermodynamic Reference Database (https://www.thereda.de). Supplementary information is available. See DOI: https://doi.org/10.1039/d5ra04721h.
