# Supplementary material for: Improved Pitzer activity model for Tc(iv) solubility and hydrolysis in the Tc(iv)–Na+–K+–Ca2+–Mg2+–H+–Cl−–OH−–H2O(l) system
Source: RSC Adv. 2025 Oct 10;15(45):37816–23. doi: 10.1039/d5ra04721h (PMC12512013; doi:10.1039/d5ra04721h)
Supplement: RA-015-D5RA04721H-s001 [file RA-015-D5RA04721H-s001.pdf]

Supplementary Information (SI) for RSC Advances.
This journal is © The Royal Society of Chemistry 2025

Supporting Information for

Improved Pitzer activity model for Tc(IV) solubility and hydrolysis in the Tc(IV)–Na^{+}–K^{+}–Ca^{2+}–Mg^{2+}–H^{+}–Cl^{-}–OH^{-}–$H_2O(l)$ system

C. Kiefer*, D. Fellhauer, M. Altmaier, X. Gaona*

Karlsruhe Institute of Technology, Institute for Nuclear Waste Disposal, Karlsruhe (Germany)

*Corresponding authors:

C. Kiefer, e-mail: christian.kiefer2@kit.edu

X. Gaona, e-mail: xavier.gaona@kit.edu

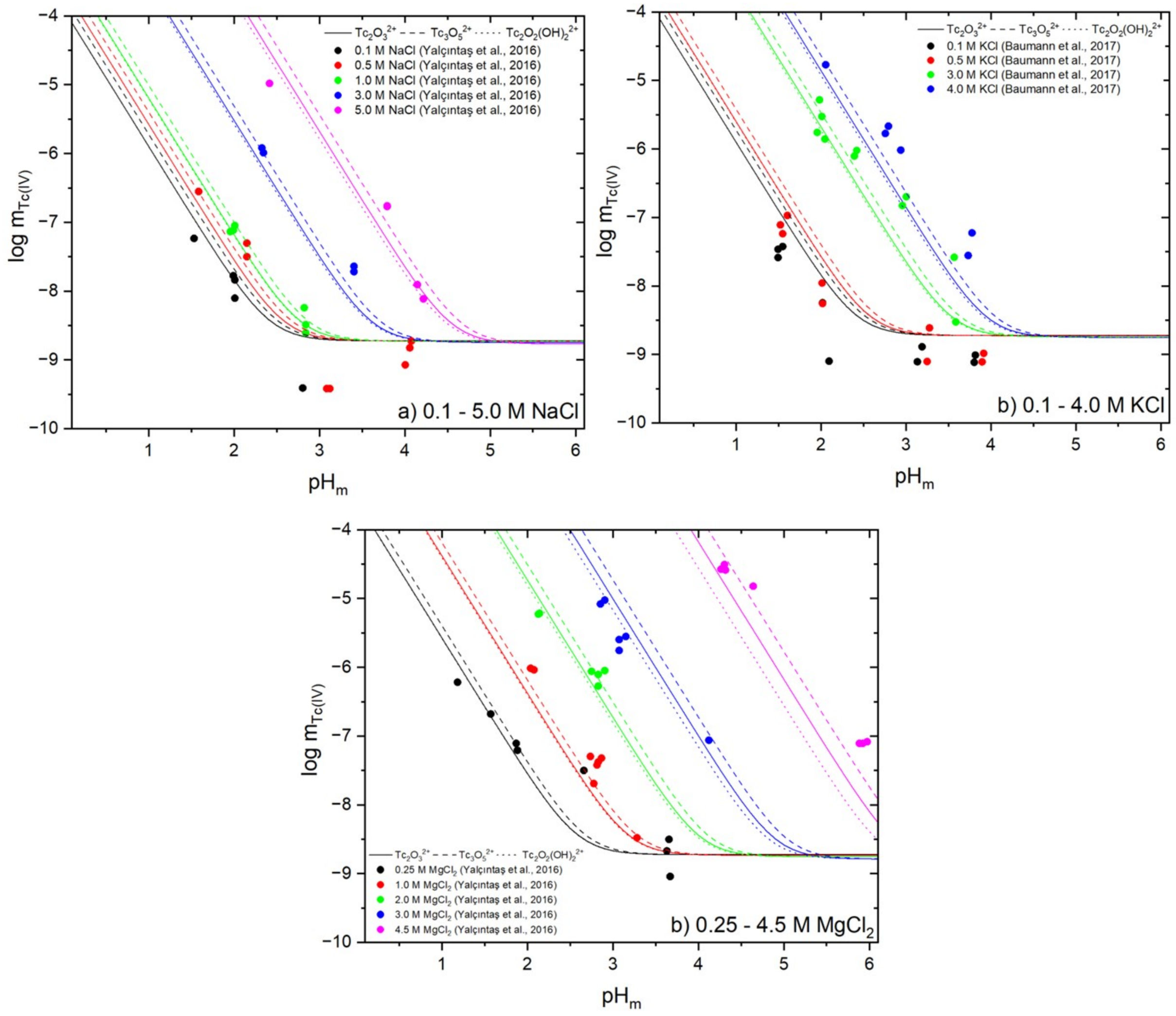

Figure SI-1: Comparison of models using the different tested acidic Tc species $Tc_2O_3^{2+}$, $Tc_3O_5^{2+}$ and $Tc_2O_2(OH)_2^{2+}$ with data in the corresponding systems.

Table SI-1: Quantification of the quality of the fits for $Tc_2O_3^{2+}$, $Tc_3O_5^{2+}$ and $Tc_2O_2(OH)_2^{2+}$, expressed as SSR and SSR/No (see text for detailed explanations).

**Species**	**$Tc_2O_3^{2+}$**	**$Tc_3O_5^{2+}$**	**$Tc_2O_2(OH)_2^{2+}$ (NEA)**
**SSR**	21.775	19.689	27.229
**No. samples (pH < 6)**	100	100	100
**SSR/No.**	0.218	0.197	0.272

Table SI-2: Quantification of the quality of the fits for alkaline NaCl and KCl systems, expressed as SSR and SSR/No (see text for detailed explanations).

**System NaCl**	
SSR	10.727
No. samples	76
SSR/No.	0.141
**System KCl**	
SSR	16.531
No. samples	91
SSR/No.	0.182

Table SI-3: Summary of binary (β^0, β^1, β^2, C^Φ), mixed electrolyte (θ_{Mc}, θ_{Xa}, Ψ_{cXa}, Ψ_{Mca}) Pitzer parameters of the background electrolyte system Na^+-K^+-Ca^{2+}-Mg^{2+}-H^+-Cl^--OH^--$H_2O(l)$ used in the present model as selected in THEREDA (Release 2023).

**Binary Pitzer parameter**						
**Ion 1**	**Ion 2**	**β^0**	**β^1**	**β^2**	**C^φ**	**Primary source**
H^+	Cl^-	0.1762	0.2995	0	0	(Voigt, 2020a)
Na^+	Cl^-	0.0754	0.2763	0	0.0015	(Voigt, 2020b)
K^+	Cl^-	0.0481	0.2181	0	-0.0008	(Voigt, 2020a)
Ca^{2+}	Cl^-	0.3065	1.7081	0	0.0022	(Voigt, 2020a)
Mg^{2+}	Cl^-	0.3523	1.6815	0	0.0052	(Voigt, 2020a)
Na^+	OH^-	0.1044	0.1246	0	0.0022	(Voigt, 2020a)
K^+	OH^-	0.1373	0.3349	0	0.0018	(Voigt, 2020a)
Ca^{2+}	OH^-	-0.1098	-0.2303	-5.72	0	(Voigt, 2020a), α^2 = 50
Mg^{2+}	OH^-	0	0	0	0	(Voigt, 2023)

**Mixed electrolyte Pitzer parameter**					
**Ion 1 (i)**	**Ion 2 (j)**	**Ion 3 (k)**	**θ_{ij}**	**Ψ_{ijk}**	**Primary source**
H^+	Na^+	Cl^-	0.0345	-0.0025	(Voigt, 2020a)
H^+	K^+	Cl^-	0.0154	-0.0132	(Voigt, 2020a)
H^+	Ca^{2+}	Cl^-	0.0969	-0.0119	(Voigt, 2020a)
H^+	Mg^{2+}	Cl^-	0.1012	-0.0099	(Voigt, 2020a)
Na^+	K^+	Cl^-	-0.0120	-0.0018	(Voigt, 2020a)
Na^+	K^+	OH^-	-0.0120	-0.0037	(Voigt, 2020a)
Na^+	Ca^{2+}	Cl^-	0.0581	-0.0011	(Voigt, 2020a)
Na^+	Ca^{2+}	OH^-	0.0581	0.2552	(Voigt, 2020a)
Na^+	Mg^{2+}	Cl^-	0.0700	-0.0120	(Voigt, 2020a)
K^+	Ca^{2+}	Cl^-	0.1156	-0.0432	(Voigt, 2020a)
K^+	Mg^{2+}	Cl^-	$9.64\cdot10^{-10}$	-0.0220	(Voigt, 2020a)
Ca^{2+}	Mg^{2+}	Cl^-	-0.0180	-0.0118	(Voigt, 2020a)
OH^-	Cl^-	Na^+	-0.0551	-0.0043	(Voigt, 2020a)
OH^-	Cl^-	K^+	-0.0551	-0.0032	(Voigt, 2020a)
OH^-	Cl^-	Ca^{2+}	-0.0551	-0.0378	(Voigt, 2020a)
OH^-	Cl^-	Mg^{2+}	-0.0551	0	(Voigt, 2020a), (Harvie et al., 1984)

References

Harvie, C.E., Moller, N., Weare, J.H., 1984. The prediction of mineral solubilities in natural waters: The Na-K-Mg-Ca-H-Cl-SO_4-OH-HCO_3-CO_3-CO_2-H_2O system to high ionic strengths at 25°C. Geochimica et Cosmochimica Acta 48, 723–751.

THEREDA - Themodynamic Reference Database. Release 2023, https://www.thereda.de/, 2024.

Voigt, W., 2023. Implementation of Carbonates and CO_2 into the T-dependent Pitzer Model of Oceanic Systems. I. System NaOH-$Mg(OH)_2$-$Ca(OH)_2$-CO_2-H_2O. Thereda Journal 03(02).

Voigt, W., 2020a. Hexary System of Oceanic Salts – Polythermal Pitzer Dataset (numerical supplement). Thereda Journal 01–01, 1–9.
Voigt, W., 2020b. Temperature extension of NaCl Pitzer coefficients and $\Delta_R G°(NaCl)$. Thereda Journal 01–02, 1–6.
